# Recombinations in Staphylococcal Cassette Chromosome *mec* Elements Compromise the Molecular Detection of Methicillin Resistance in *Staphylococcus aureus*

**DOI:** 10.1371/journal.pone.0101419

**Published:** 2014-06-27

**Authors:** Grant A. Hill-Cawthorne, Lyndsey O. Hudson, Moataz Fouad Abd El Ghany, Olaf Piepenburg, Mridul Nair, Andrew Dodgson, Matthew S. Forrest, Taane G. Clark, Arnab Pain

**Affiliations:** 1 Pathogen Genomics Laboratory, King Abdullah University of Science and Technology (KAUST), Thuwal, Saudi Arabia; 2 Marie Bashir Institute for Infectious Diseases and Biosecurity and Sydney School of Public Health, University of Sydney, Sydney, Australia; 3 Department of Infectious Disease Epidemiology, Imperial College London, London, United Kingdom; 4 TwistDx Ltd., Babraham, United Kingdom; 5 Microbiology Dept, Central Manchester University Hospitals NHS Foundation Trust, Manchester, United Kingdom; 6 Department of Infectious Disease Epidemiology, London School of Hygiene and Tropical Medicine, London, United Kingdom; University Hospital Münster, Germany

## Abstract

Clinical laboratories are increasingly using molecular tests for methicillin-resistant *Staphylococcus aureus* (MRSA) screening. However, primers have to be targeted to a variable chromosomal region, the staphylococcal cassette chromosome *mec* (SCC*mec*). We initially screened 726 MRSA isolates from a single UK hospital trust by recombinase polymerase amplification (RPA), a novel, isothermal alternative to PCR. Undetected isolates were further characterised using multilocus sequence, *spa* typing and whole genome sequencing. 96% of our tested phenotypically MRSA isolates contained one of the six *orfX*-SCC*mec* junctions our RPA test and commercially available molecular tests target. However 30 isolates could not be detected. Sequencing of 24 of these isolates demonstrated recombinations within the SCC*mec* element with novel insertions that interfered with the RPA, preventing identification as MRSA. This result suggests that clinical laboratories cannot rely solely upon molecular assays to reliably detect all methicillin-resistance. The presence of significant recombinations in the SCC*mec* element, where the majority of assays target their primers, suggests that there will continue to be isolates that escape identification. We caution that dependence on amplification-based molecular assays will continue to result in failure to diagnose a small proportion (∼4%) of MRSA isolates, unless the true level of SCC*mec* natural diversity is determined by whole genome sequencing of a large collection of MRSA isolates.

## Introduction

*Staphylococcus aureus* is an important human pathogen and is responsible for healthcare-, community- and livestock-associated colonisation and infection [1]. Infections are especially problematic if the bacteria are methicillin-resistant *S. aureus* (MRSA), which also exhibit phenotypic resistance to related β-lactam antibiotics including flucloxacillin, cefoxitin and oxacillin. As such there is a great demand for rapid tests to detect MRSA.

Methicillin-sensitive *S. aureus* (MSSA) becomes MRSA when it acquires the SCC*mec* (staphylococcal cassette chromosome *mec*) genetic element. This element contains the *mec* gene that encodes the protein PBP2A (penicillin binding protein 2A). Co-colonising coagulase-negative staphylococci (CNS) are thought to act as the reservoir for the *mec* gene with *S. fleurettii* suggested to be the origin [2]. CNS species are commensal human skin organisms but have been found to act as a reservoir for entire SCC*mec* elements [3]. Co-colonisation of CNS carrying *mec* elements and MSSA means that nucleic acid tests (NATs) for *mecA* and an MSSA-specific gene can give false positive results. NATs for MRSA therefore need to specifically detect the insertion of the SCC*mec* element into the *orfX* gene of *S. aureus.* This testing is performed by amplifying with a primer in the SCC*mec* element and a primer in the *orfX* gene of the *S. aureus* chromosome [4]. Clinical microbiology laboratories are increasingly turning towards such molecular testing for the first-line identification of MRSA carriage. However, a recent systematic review concluded that there is insufficient evidence on the clinical effectiveness of PCR over other hospital MRSA screening methods [5]. In particular, a growing number of *S. aureus* isolates are phenotypically resistant but produce false negative results using currently marketed real-time PCR assays [6]–[9]. Sensitivities for currently marketed PCR assays are as low as 69% compared to culture [10], resulting in MRSA isolates being misidentified as MSSA [11], [12]. Some of the false negatives are likely due to the diversity of the *orfX*-*SCCmec* junctions, as to date at least twenty types have been identified worldwide [13]. This variability means that a unique primer must be developed for each novel *orfX*-*SCCmec* sequence if it is to be detected. The prevalence of different junction types is not well known, but it is suggested that types i, ii, iii, iv, v and vii, account for over 98% of worldwide strains tested [4]. We decided to investigate if this was true at a large UK hospital by screening several hundred MRSA isolates using a Recombinase Polymerase Amplification (RPA) multiplex assay.

RPA is a novel, isothermal nucleic acid amplification chemistry [14]. The need for complex thermal cycling instruments for PCR is replaced by three core enzymes that operate optimally at 37–40°C. The first enzyme, a recombinase, binds to primers, forming filaments that can then recombine with homologous DNA. The second enzyme, a single-stranded DNA binding protein, binds to the strand of DNA that is displaced by the primer, preventing the dissociation of the primer. The final core enzyme is a strand-displacing polymerase that copies the DNA, adding bases onto the 3′ end of the primer, forcing open the DNA double helix as it progresses. When opposing primers are used, exponential amplification occurs, with reactions typically running to completion in 5–20 minutes depending upon amplicon size and starting template copy number. Real-time fluorescent detection of RPA reactions is achieved with TwistAmp exo probes. These feature an internal fluorophore and quencher a few bases apart, with an intervening abasic site (tetrahydrofuran, THF). If the TwistAmp exo probe binds to a complementary sequence then this THF becomes a substrate for exonuclease III and is cleaved, separating fluorophore and quencher. As amplicon is generated, increasing numbers of probes are cleaved, typically giving a detectable signal in 5–10 minutes. RPA reactions are provided as stable, lyophilised pellets that contain all of the necessary enzymes and reagents (www.twistdx.co.uk).

As the price of whole-genome sequencing fell significantly after our initial study, it became possible to expand the scope of our investigation and use whole genome sequencing to further identify the precise reasons why our RPA multiplex failed to detect some of the isolates as MRSA.

Failing to identify carriage of MRSA in hospital inpatients will have significant consequences for the individual patient and for general infection control. With the high variability in both *orfX*-SCC*mec* junctions and the sequences of SCC*mec* elements circulating in hospitals it remains to be seen if one assay will be effective as a screening tool. Rapid whole-genome sequencing has revolutionised the investigation of MRSA outbreaks and transmission [15], [16] but it can also aid us in identifying isolates that cannot be detected with current molecular assays and enable researchers to alter their tests to detect them.

## Methods

### Strains

Central Manchester University Hospitals NHS Foundation Trust (CMFT) is a large academic trust comprising of specialist hospitals for children, dentistry and ophthalmology, together with a large teaching hospital and community services. MRSA isolates were obtained from clinical samples collected via standard hospital screening procedures from the Trust. A total of 726 isolates were collected from 726 patients at CMFT between December 2008 and June 2009 (n = 580) and between July 2009 and November 2009 (n = 146).

Isolates were first screened using a multiplex RPA test. The CMFT standard screening procedure was to collect nasal and groin swabs and combine to inoculate 15 ml nutrient broth (Oxoid, CM1) containing 7.5% NaCl. Broths were incubated for a minimum of 18 hrs at 35°C in air and then subcultured on MRSA Select agar plates (BD Diagnostics). In addition to usual diagnostics, a single, pink, colony was picked from each positive plate after 24 hours incubation, streaked onto an anonymised blood agar plate and incubated for a further 24 hours. A lack of suitable biocontainment facilities at TwistDx meant that plates were scraped and bacteria resuspended and boiled for 20 minutes. Lysed bacteria were then diluted 1∶1000 in sterile distilled water for use in RPA reactions.

### Recombinase polymerase amplification

RPA primers differ from PCR only in length, with 30–38 bases being optimal for efficient recombinase filament formation. TwistAmp exo probes are typically 46–52 bases long, with a THF ≥30 bases from the 5′ end and ≥15 bases from the 3′ end. A fluorophore and a quencher are positioned either side of the THF such that cleavage by exonuclease III separates the two and fluorescence increases. TwistAmp exo probes have a C3-spacer or similar block at their 3′ end to prevent them amplifying DNA unless cleaved. Numerous overlapping primers were tested empirically with 25 copies of PCR product for each *orfX*-SCC*mec* junction type to determine the best combinations for multiplexing. Potential confounding SNPs were identified by BLAST searches and oligonucleotides targeted to the most conserved regions of each junction. *OrfX* was compared to CNS using BLAST to identify the most divergent sequences from *S. aureus* to minimise the risk of false positives caused by amplification of methicillin resistant CNS. To determine the range of *SCCmec* element types that TwistAmp MRSA was able to detect, 15 prototypic strains (Table S1) for *SCCmec* types I-XI were tested with the assay [17]. TwistAmp MRSA was able to detect 11, representing *SCCmec* types I-IV and VI-VIII. The *SCCmec* type V prototype strain (WIS) was not detected by TwistAmp MRSA and was later identified as containing a type of junction, xii, not covered by the assay. Performing a BLAST alignment of all *SCCmec* type V entries in GenBank other than WIS (both types 5C2 and 5C2&5 accession numbers AB505629, AM990992, GQ902038, FJ830606, AB478780, AB512767, AB462393 and CP003166) revealed that they all contained type iii junctions, suggesting that they would be successfully detected by the multiplex.

The selected oligonucleotides were tested for specificity with 10^6^ copies of genomic DNA from *S. saprophyticus* (ATCC 43867), *S. epidermidis* (ATCC 35983) and *S. hominis* (ATCC 51624) and gave no signal. We developed a multiplex RPA reaction that detected, but did not differentiate, junctions i, ii, iii, iv, v and vii. Isolates that were positive by this test were typed by uniplex assays for junction ii, i, iii, iv, v and vii. 50 µl RPA reactions pellets that included the primers and probes were freeze-dried by TwistDx Ltd, Babraham, Cambridge. For the multiplex reaction an internal control sequence was also included to confirm that the reactions had worked. This internal control DNA was designed with the junction i and iii primers at opposite ends with the sequence detected by the control probe in between (Table 1).

**Table 1:** Primers and TwistAmp exo probes used in multiplex and singleplex RPA reactions to identify *orfX*-SCC*mec* junction types in MRSA isolates.

Oligonucleotide	Nucleotide sequence 5′–3′	Reference sequence	Nucleotides (5′-3′)
mrej-i	CTGCGGAGGCTAACTATGTCAAAAATCATGAACCTCAT	AB033763.2	38813..38850
mrej-ii	ACAGCAATTCACATAAACCTCATATGTTCT	BA000018.3	34244..34215
mrej-iii	ATGTAATTCCTCCACATCTCATTAAATTTTTAAAT	AB037671.1	67719..67753
mrej-iv	TCCATCTCTACTTTATTGTTTTCTTCAAATATT	AY267374.1	539..507
mrej-v	AACTCTGCTTTATATTATAAAATTACGGCTGAAA	AY267381.1	489..466
mrej-vii	TTCACTTTTTATTCTTCAAAGATTTGAGCTAATTT	AY267375.1	531..497
orfX	CAACGCAGTAACTATGCACTATCATTTAGCAAAAT	AY267375.1	346..380
orfX	CAACGCAGTAACTACGCACTATCATTCAGCAAAAT	BA000018.3	34046..34080
orfX-probe	CATTCCCACATCAAATGATGCGGGTTGTGT12A3TGARCAAGTGTA	BA000018.3	34083..34128
Internal control-probe	CGATCATGCCCATCAGCAGCTTATGATCAA425GATCCAAACCGAGGCG	N/A	N/A

IUPAC ambiguity codes are used where necessary. Non-standard bases are as follows: 1 = dT FAM; 2 = tetrahydrofuran; 3 = dT Black Hole Quencher (BHQ) 1; 4 = dT TAMRA; 5 = dT BHQ2. BHQ available from Biosearch Technologies, Novato, CA.

RPA reactions were performed by adding 49 µl MRSA resuspension buffer and 1 µl of the boiled, diluted MRSA isolate. Once a strip of 8 reactions had been resuspended, lids were placed on the 0.2 ml PCR tubes and the strip vortexed and pulse-spun. Reactions were run for 20 minutes in Twista portable real-time fluorometers pre-heated to 39°C (Figure S1). Strips were removed, vortexed and pulse-spun and replaced after 4 and 6 minutes to agitate the reactions.

Isolates that were positive by the RPA *orfX*-*SCCmec* junction multiplex were then tested using uniplex RPA reactions to individual *orfX*-*SCCmec* junction targets. Isolates that were negative by the RPA *orfX*-*SCCmec* junction multiplex were sent for MLST, *spa* typing and high-throughput sequencing.

### Further molecular characterisation

We used the MLST scheme previously developed [18] to characterise the isolates on the basis of the sequences of seven housekeeping genes (*arc, aroE, glpF, gynK, pta, tpi and yqiL*). The sequence type (ST) of each isolate was determined using the online MLST database (http://saureus.mlst.net/). eBURST (http://eburst.mlst.net/) was used to determine founding genotypes. The *spa* type of each isolate was determined using methods described previously [19].

### Review of MRSA SCC*mec* sequences available on GenBank

57 high quality annotated sequences of MRSA SCC*mec* regions were found by searching the GenBank Nucleotide database (February 2013). Features were extracted and manually curated into a protein fasta file and analysed using OrthoMCL v2.0. Maximal discrimination between similar proteins was achieved by using an inflation parameter (I) of 13. OrthoMCL could not distinguish between different Ccr and Mec allotypes due to their high amino acid similarity.

### High-throughput sequencing and assembly

Extracted DNA from isolates undetectable by RPA to the *orfX*-*SCCmec* junction were run on an E-Gel 2% gel System (Invitrogen) and examined for quality of DNA and degree of degradation. DNA from 24 (80%) of the 30 isolates was judged to be of sufficient quantity and quality for library making and sequencing. Unfortunately the other six isolate cultures were unrecoverable and so further DNA could not be sourced. The isolates were sequenced on an Illumina Genome Analyzer II, with 101 base pair reads and a paired-end insert size of 400 bp to an average coverage of 100-fold. It is regrettable that we were not able to sequence all 30 undetected isolates and that culturable isolates are not available for them. When this study was started the cost of whole-genome sequencing was prohibitive and it was only the subsequent precipitous drop in cost that allowed us to re-evaluate what it was possible to do with the samples that we had collected. The sequence reads were trimmed and corrected by Phred+33 quality values using Quake [20]. *De novo* sequence assembly was carried out using Velvet [21] with the optimal kmer size ranging from 33 to 75. Sequencing contigs and scaffolds were first automatically rearranged and ordered using ABACAS [22] and then homology with existing SCC*mec* types in GenBank determined using BLASTN on a custom database. Manual reordering was performed by visualisation of BLAST results in ACT [23]. Where the *orfX*-*SCCmec* junction was not fully assembled 20–30 iterations of IMAGE [22] were performed. SCC*mec* types were annotated using RATT [22] before undergoing manual annotation using Artemis [24]. All annotated SCC*mec* sequences have been uploaded to GenBank [accession numbers HF569093–HF569116]. Unfortunately frozen stocks of these bacteria were no longer available at CMFT by this time. With the sequence information that we have published however, researchers will be able to order synthetic versions of novel junction regions with which to test any new assays that they may wish to develop.

## Results

### Recombinase polymerase amplification

Of the 726 MRSA isolates tested, 696 (96%) could be detected by *orfX*-*SCCmec* junction multiplex RPA. *orfX*-*SCCmec* junction uniplex-RPA testing showed that most (653) of these 696 isolates were *orfX*-*SCCmec* junction-ii, with the other *orfX*-*SCCmec* junction types tested only representing a minority of cases (i and iii, 1.8%; iv, 0.3%; v, 0.1%; vii, 1.9%).

### Comparison of MRSA SCC*mec* sequences in GenBank

To understand better the conservation of proteins in MRSA, the proteins from the 57 MRSA SCC*mec* sequences available in GenBank were compared using OrthoMCL (Figure S2). A core group of proteins appear in most SCC*mec* elements with the most frequent occurring proteins being *mec* (100% strains), an uncharacterised protein (OG63_2, 89.5%), IS*431* transposase (89.5%), *ugpq* (87.7%), *ccrA* (84.2%), *ccrB* (82.5%) and *maoC* (82.5%). Strains clustered based on the SCC*mec* types proposed by the International Working Group on the Classification of Staphylococcal Cassette Chromosome Elements (IWG-SCC) [25].

### Further molecular characterisation

**Figure 1:**
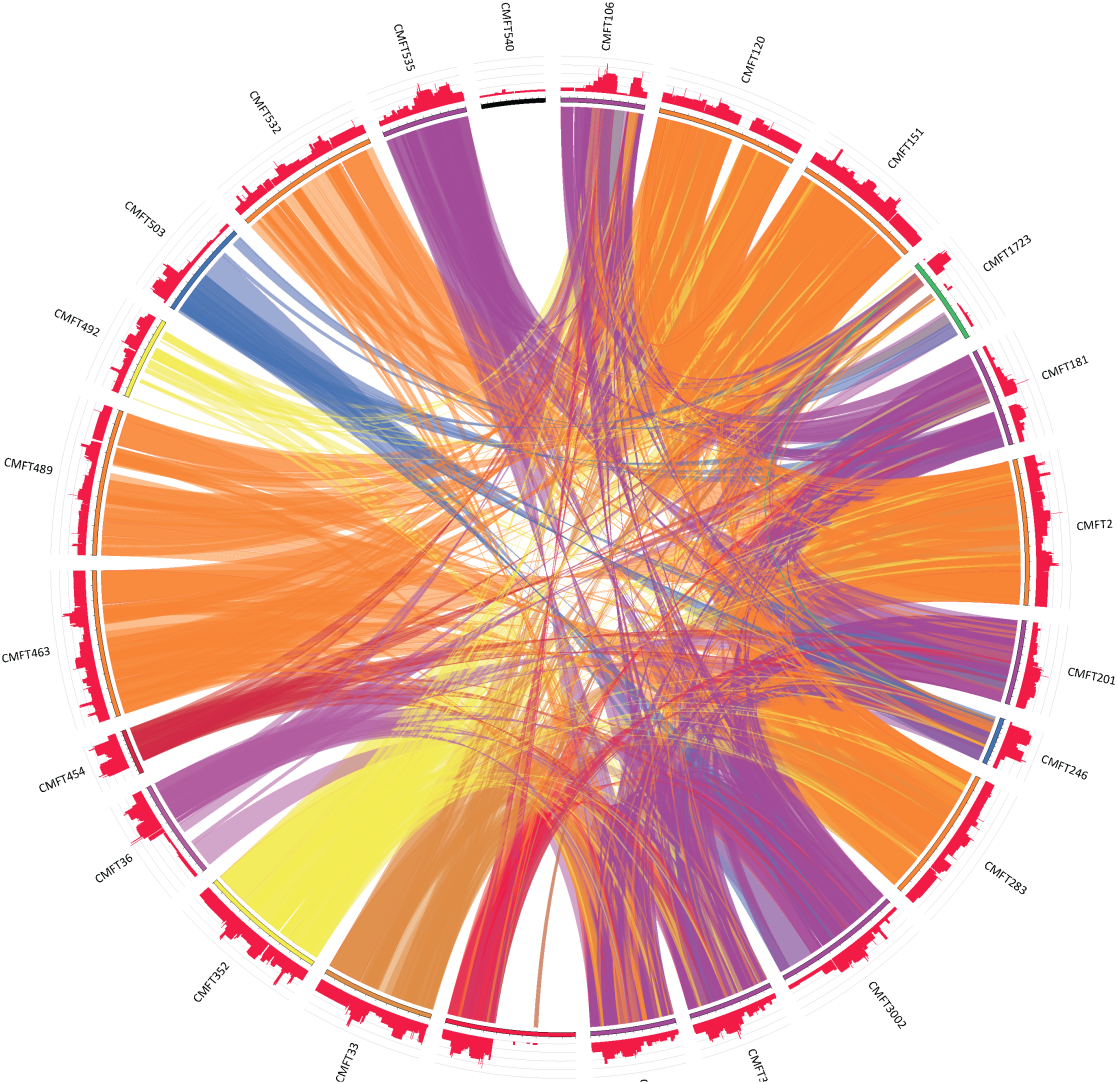
Level of homology between 24 sequenced SCC*mec* elements using the Circos tool [52]. All-against-all BLASTN using E value of 10^−300^ as cut-off. 4738 local alignments produced in total, internal ribbons show 2465 alignments to preserve clarity. Histograms around circumference of circle show distribution of all 4738 alignments. Colours correspond to SCC*mec* type: red, IVh-CMFT3119; light purple, IVj; blue, IVa; purple, IVk; orange, IIa-CMFT2-like; yellow, IIa-CMFT492-like; dark orange, II.5; dark red, IVe; black, XI; green, V. Very little homology seen for CMFT540 (type XI) and region of CMFT3119 containing *arc* gene complex and *ccr*C.

DNA from the original extract was available for 28 of the 30 *orfX*-*SCCmec* isolates for which the junction was undetectable by RPA and these underwent further analysis using MLST and *spa* typing. Only 24 of the isolates were recoverable for further DNA extraction for second-generation sequencing. Unfortunately, because isolates had been anonymised and the cultures taken from them boiled, it was not possible to return to CMFT and re-grow any strains that had poor quality DNA. Table 2 shows the results of the distribution of ST, *spa* and SCC*mec* types (where available) within these 28 isolates. Eight STs in total were found, none of which were closely related except for ST30, which is a single locus variant (SLV) of ST36. 18 *spa* types were found among the 28 isolates tested. Sequencing of the SCC*mec* elements demonstrates conserved homology for many of the isolates but with one cassette displaying no homology and variable levels among the others (Figure 1). The elements identified by sequencing were reviewed by the IWG-SCC. As the elements are conjugates the SCC and SCC*mec* element subtypes have been assigned. When a novel conjugate element was described the first isolate that was found in our set becomes the archetype and elements with the same structure are called CMFTx-like for simplicity in the text.

**Table 2:** Further characterisation of isolates undetectable by recombinase polymerase amplification: MLST and *spa* typing for the 28 isolates and sequencing data for the 24 isolates for which DNA could be recovered.

Isolate number	ST^a^	Clonal complex (CC)^b^	*spa* type	SCC*mec* c type	Homology to SCCmec (accession no.)	Main changes	Reason for lack of RPA result
CMFT109	15	15	t084	*	*	*	*
CMFT3119	22	22	t025	IVh-CMFT3119-like	ZH47 (AM292304) M1 (HM030720)	*+ arc* complex Substitution*ccr* 4 for 5	Multiple sites for primer binding
CMFT36	22	22	t032	IVj	JCSC6670 (AB425824)	+ *hsd* complex	Fragment too large
CMFT246	22	22	t223	IVa (–ACME)	USA300 (NC_007793)	− ACME	Too many primer mismatches
CMFT503	22	22	t309	IVa (–IS431)	JKD6159 (CP002114)	+ CDS at IS431	Too many primer mismatches
CMFT201	22	22	t906	IVk	45394F (GU122149)	− *hsd* complex	Fragment too large
CMFT211	22	22	t906	*	*	*	*
CMFT303	22		t906	IVk	45394F (GU122149)	− *hsd* complex	Fragment too large
CMFT306	22		t6420	IVk	45394F (GU122149)	− *hsd* complex	Fragment too large
CMFT535	22		t6421	IVk	45394F (GU122149)	− *hsd* complex	Fragment too large
CMFT432	30	30	t017	*	*	*	*
CMFT2	36	30	t018	IIa-CMFT2-like	MRSA252 (NC002952)	+ class 1 *ccr*	Too many primer mismatches
CMFT120	36	30	t018	IIa-CMFT2-like	MRSA252 (NC002952)	+ class 1 *ccr*	Too many primer mismatches
CMFT151	36	30	t018	IIa-CMFT2-like	MRSA252 (NC002952)	+ class 1 *ccr*	Too many primer mismatches
CMFT283	36	30	t018	IIa-CMFT2-like	MRSA252 (NC002952)	+ class 1 *ccr*	Too many primer mismatches
CMFT463	36	30	t018	IIa-CMFT2-like	MRSA252 (NC002952)	+ class 1 *ccr*	Too many primer mismatches
CMFT489	36	30	t018	IIa-CMFT2-like	MRSA252 (NC002952)	+ class 1 *ccr*	Too many primer mismatches
CMFT532	36	30	t018	IIa-CMFT2-like	MRSA252 (NC002952)	+ class 1 *ccr*	Too many primer mismatches
CMFT492	36	30	t018	IIa-CMFT492-like	MRSA252 (NC002952)	+ *ccr* 1/− puB110/− Tn*554*	Too many primer mismatches
CMFT352	36	30	t018	IIa-CMFT492-like	MRSA252 (NC002952)	+ *ccr* 1/− puB110/− Tn*554*	Too many primer mismatches
CMFT33	36	30	t021	II.5	MRSA252 (NC002952)	pUB110 inverted	Two fragments produced
CMFT454	59	59	t216	IVE	AR43/3330.1 (AJ810121)	Minor J1 changes	Multiple sites for primer binding
CMFT374	59	59	t6419	*	*	*	*
CMFT540	130	130	t843	XI	LGA251 (FR821779)	No changes	Too many primer mismatches
CMFT106	149	5	t5626	IVk	45394F (GU122149)	− *hsd* complex	Fragment too large
CMFT181	149	5	t5181	IVk	45394F (GU122149)	− *hsd* complex	Fragment too large
CMFT3002	149	5	t5829	IVk	45394F (GU122149)	− *hsd* complex	Fragment too large
CMFT1723	772	1	t657	V	WIS (AB121219)	+ pepF (SAR1397)	Too many primer mismatches

*Lack of good quality DNA for sequencing.

a Sequence type.

b As determined by Multi-Locus Sequence Typing (MLST).

c Staphylococcal cassette chromosome *mec.*

Reasons for the negative RPA result for the sequenced isolates are given.

### Changes in SCC*mec* type II in ST36 isolates

**Figure 2:**
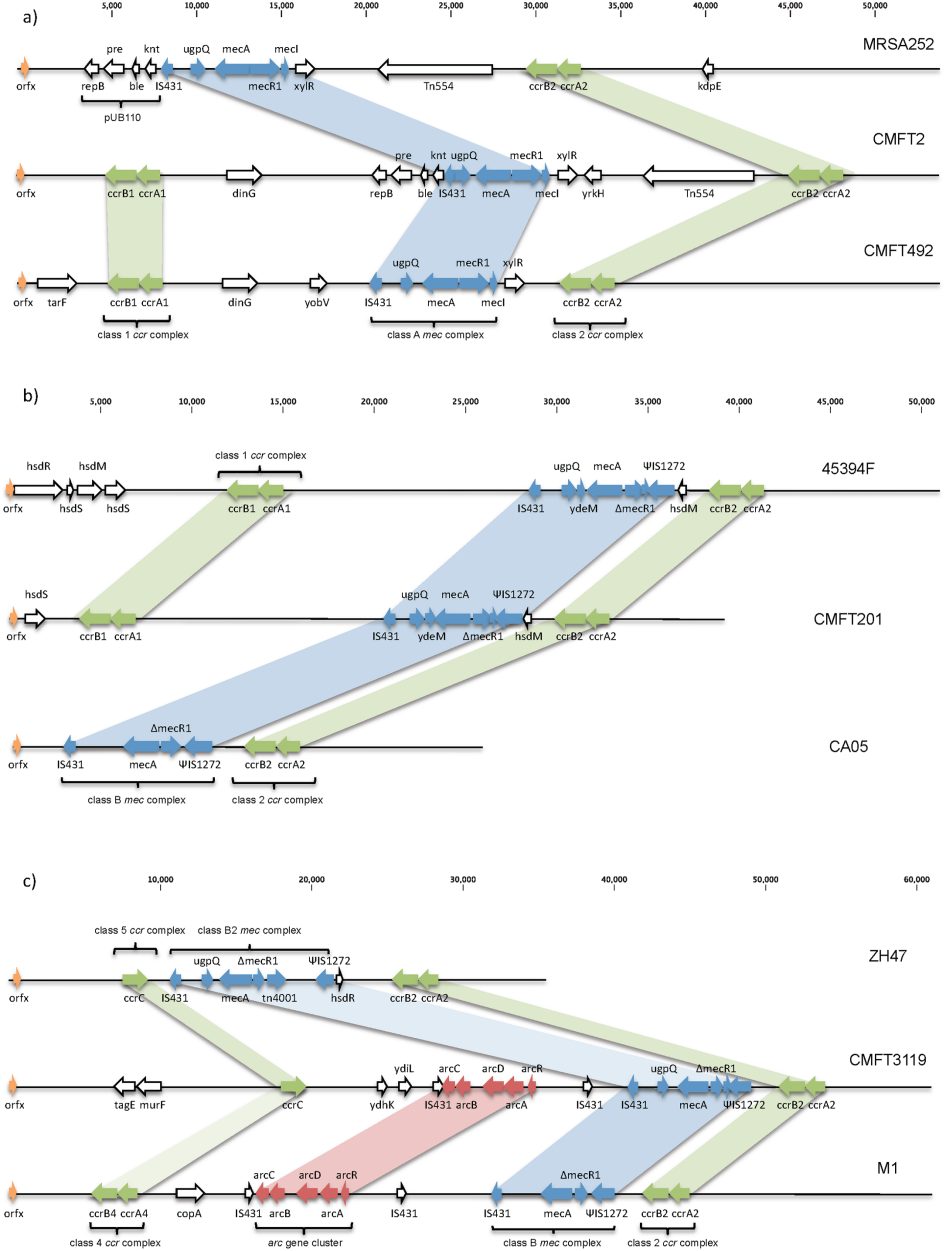
Variant SCC*mec* elements. **a)** SCC*mec* for archetypal type II, MRSA252 (NC_002952), compared to isolates CMFT2 and CMFT492. The cassette of CMFT2 shows the typical features of a type II SCC*mec*; a class A *mec* complex and a class 2 *ccr* complex. However, there is an additional SCC carrying type I *ccr* genes situated at the 5′ end of the element. CMFT492 is superficially similar and contains the same additional class 1 *ccr* complex. However, it lacks two of the major features of most type II SCC*mec* elements; the plasmid vector pUB110 and transposon Tn*554*. **b)** SCC*mec* type IVk: SCC*mec* for CMFT201 compared to CA05 (AB063172, type IV(2B)) and 45394F (GU122149). The cassette of CMFT201 shows the typical features of a type IV SCC*mec* (CA05); a class B *mec* complex and a class 2 *ccr* complex. However, there are additional SCC elements with an SCC carrying type I *ccr* genes situated at the 5′ end of the cassette. This is a similar structure to that shown by strain 45394F (unpublished). **c)** Type IVh variant SCC-SCC*mec* element for CMFT3119 compared to strains ZH47 (AM292304) and M1 (HM030720). The cassette of CMFT3119 shows the typical features of a type IV SCC*mec*; a class B *mec* complex and a class 2 *ccr* complex. However CMFT3119 contains an additional SCC carrying a *ccrC* gene upstream of the *mec* complex, similar to the recombination seen in ZH47. In contrast to ZH47 there is not a Tn*4001* but instead part of the arginine catabolic mobile element (ACME), seen in *S. epidermidis, S. haemolyticus* and USA300, has been inserted. This *arc* gene cluster is very similar to that seen in the recently identified strain M1.

The major MLST groups were 22 (9 isolates) and 36 (10 isolates). ST36 belongs to clonal complex 30 (CC30). All, but one of the isolates were *spa* type t018. One isolate was characterised as ST30, an SLV of ST36. Sequencing of the ten ST36 isolates demonstrated that their SCC*mec* elements were all type II as expected within this group, but with significant changes that prevented the RPA assay from working (Table 2). Seven of the isolates contain the same conjugate element, of which CMFT2 is an example (Figure 2a). This cassette has near identical gene order to the type II reference genome MRSA252 [26]; with a class 2 *ccr* complex and class A *mec* complex, pUB110 and Tn*554* (Figures 2a and S3a). As well as the SCCmec element there is an additional SCC carrying type I *ccr* genes close to the 5′ end of the element (Figures 2a and S3b). No such composite elements with this pattern are present in GenBank. Two isolates have a variant of this element, which have been labelled IIa-CMFT492-like. This has the same structure but lacks the pUB110 plasmid vector and Tn*554* transposon (Figure 2a).

### Changes in SCC*mec* type IV in ST22 isolates

ST22-MRSA-IV is the pandemic strain known as UK-EMRSA-15. In the UK ST22-MRSA-IV has become increasingly common, at the expense of ST36-MRSA-II [27], the other predominant group among the *orfX*-*SCCmec* junction-undetectable isolates. It is now responsible for 85% of MRSA bacteraemia cases in UK hospitals. Nine isolates belong to CC22, a common and widespread group that carries SCC*mec* type IV. Eight ST22-MRSA-IV strains were sequenced and four of them had elements that have a different structure to previously published type IV cassettes. CMFT201 shows the characteristic type IV features of a class 2 *ccr* gene complex and class B *mec* gene complex. However, similar to the variant type II elements discussed above, there is a further class 1 (A1B1) *ccr* complex situated at the 5′ end of the cassette (Figure 2b). Comparison to all SCC*mec* elements available in GenBank demonstrates remarkable homology to an unpublished strain 45394F (GU122149) (Figures 2b and S4a). The key differences in homology are at the 5′ end (Figure S4b) with the CMFT201 strain lacking genes *hsdR* and *hsdM*. As type I restriction and modification systems are encoded by all three genes; *hsdR*, *hsdM*, and *hsdS*; this may indicate an inability to synthesise R2M2S1 that usually modifies hemimethylated DNA and restricts unmethylated DNA [28].

An additional novel variant is only demonstrated by CMFT3119. The sequenced SCC*mec* element shares partial homology with two existing strains; ZH47 [29], and strain M1 (Figures 2c and S5) [30]. Similar to ZH47 it contains an additional *ccr*C along with the normal class 2 *ccr* gene complex. The larger SCC*mec* element in CMFT3119 is due to the addition of an *arc* gene cluster. Therefore overall CMFT3119 also bears similarity to isolate M1, which also has a class B *mec* complex, class 2 *ccr* complex and part of the arginine catabolic mobile element (ACME) often found in *S. epidermidis* and *S. saprophyticus*. This may be a subtype of IVh as the J1 region bears homology to ST22-MRSA-IVh, and some isolates belonging to this genotype can contain a truncated ACME element [31].

The three further isolates sequenced from this clonal complex had smaller changes but of a great enough magnitude to still prevent molecular detection with RPA (Table 2).

### SCCmec IVk also seen in ST149 isolates

The three isolates belonging to ST149, part of CC5, also have a type IVk SCC*mec* element. ST149 has previously been described in Malta where it appears to be common [32] and in a Libyan patient in Switzerland [33]. This clonal complex has previously been characterised as carrying multiple composite SCC*mec* elements [29]. As with the ST22-MRSA-IVk isolates these bore significant homology to 4539F but with deletion of the *hsd* complex.

### Small numbers of ST59, ST130 and ST772 also found

The two ST59 isolates belong to the major community-associated CC59 lineage, a clonal group that has become widespread in the Asia-Pacific region. CC59 strains have been described in several countries including the UK [34]–[36]. Enough DNA was available for one of the two isolates to be sequenced with a type IVE element evident. However multiple RPA primers bind due to similarities to both *orfX*-*SCCmec* junction types iv and v, leading to no clear amplified fragment generation.

CMFT540 belongs to the clonal lineage CC130 with *spa* type t843, previously reported in bovine and more recently in humans in the UK, Denmark, Ireland and Germany [37]–[39]. Sequencing confirms that this also has a type XI element with very close homology to that of LGA251 [37].

The single ST772-t657 isolate is known as the Bengal Bay clone or WA MRSA-60, a multiply-resistant Panton-Valentine Leukocidin-positive CA-MRSA that is becoming increasingly prevalent in India, where it has spread into hospitals [40]. The type V element identified by sequencing was very similar to that of WIS (AB121219) except for an additional *pepF*, more commonly found in type II cassettes.

## Discussion

Molecular methods for the detection of MRSA that are currently on the market make an attractive alternative to the slower methods of using chromogenic selective MRSA agar or by enrichment in a 7.5% NaCl nutrient broth [41]–[45]. However, the inherent weakness of all molecular tests is that they can only amplify sequences that they have been designed to detect. For many pathogens it is simple to identify conserved, signature sequences to target. However, in order to be confident that all MRSA cases are detected, a high sensitivity for all SCC*mec* element types is required. MRSA SCC*mec* elements display a high level of protein diversity resulting from significant nucleotide diversity within shared proteins. Despite this we have shown that an RPA assay can be used to assess the prevalence of the *orfX*-*SCCmec* junction types described by Huletsky et al, in a large UK teaching hospital [4]. 96% of bacteriologically confirmed MRSA isolates were detectable with the RPA assay. However, this does mean that, if used as a diagnostic assay, 30 MRSA isolates would have been false negatives. This is similar to the sensitivity rates seen for other molecular assays [46].

A previous study at a UK hospital found the majority of molecularly characterised MRSA isolates carry a type IV SCC*mec* element [47] but there is little information available for cassette distributions for the whole country. In CMFT 90% of all isolates were found to be *orfX*-*SCCmec* junction type ii. Although *orfX*-*SCCmec* junction types and SCC*mec* types do not easily correlate, *orfX*-*SCCmec* junction type ii usually corresponds to SCC*mec* element types I, II or IVd [4].

With the increased interest in molecular-only MRSA screening, missing 30 resistant isolates during screening has significant health risks. It is only in recent years that we are starting to appreciate fully the array of SCC*mec* elements that may occur. PCR and RPA primers are therefore being developed for a genetically highly variable location. It has also been difficult to identify composite SCC-SCC*mec* elements as they would either produce a large number of PCR fragments that were difficult to interpret or have a very unusual hybridisation pattern [35]. Whole-genome sequencing followed by re-mapping does not work for genome regions displaying high levels of variation. No assembled reference sequences containing all SCC*mec* elements exists which leads to very low mapping coverage (Figures S6a and b). We have demonstrated that only *de novo* assembly allows the cassette to be fully characterised (Figure S6c).

The SCC*mec* element sequences give clues as to why the RPA assay failed to detect them. For the type IVk the insertion of the additional class 1 *ccr* complex leads to a 950 bp fragment being produced by the RPA primers; too large for an RPA assay optimised for amplicons of less than 300 bp. Whilst it is possible to optimise RPA to amplify longer fragments, amplification time is a function of recombination rate and amplicon length, so it is unlikely that such a large fragment could be detected with the desired sensitivity in <15 minutes. It is likely that this represents a degree of cross-species exchange and recombination as the additional *ccr* locus is very similar to that seen in a Chinese isolate of *S. haemolyticus* [48].

Although previously recognised for its predilection for recombination, we have shown that the acquisition of composite SCC-SCC*mec* elements is not unique to ST149. Both of the most common MLST groups in UK hospitals, 36 and 22, were present amongst the RPA-undetectable isolates. Many of the ST36 isolates had a IIa cassette that had acquired an additional class 1 *ccr* complex from an SCC. In addition, two isolates had lost the puB110 and Tn*554* seen in MRSA252. Although phenotype data was not available it is likely that the loss of the bleomycin- and kanamycin-resistance genes, and erythromycin- and spectinomycin-resistance genes, from the puB110 and Tn*554* respectively, will have led to an MRSA strain susceptible to most non-methicillin antibiotics.

The composite SCC-SCC*mec* element of CMFT3119 showed a different arrangement to all of the other isolates. The variant IVh cassette was similar to that of ZH47 by having an additional class 5 *ccr* complex but it also contained an *arc* cluster like strain M1 [30]. *arc* gene complexes, or ACMEs, are common among ST8-MRSA-IVa (USA300) isolates but not often seen outside of this clonal group for MRSA. However, they are seen more commonly in coagulase negative staphylococci. *arc* and *opp* genes are homologs of genes that are recognised bacterial virulence factors and encode an arginine deaminase pathway and ABC transporter systems respectively. The *speG* gene of ACME has also been shown to be a potential virulence factor by allowing the bacterium to circumvent polyamine hypersensitivity [49]. A native *arc* cluster can be found on the chromosome of *S. aureus* but the ACME-*arc* cluster inserts as an SCC-like element adjacent to the SCC*mec* in some strains. Animal models have suggested that the presence of ACME clusters in ST8-MRSA-IVa strains leads to an improved fitness and ability to colonise the skin and mucous membranes [50], [51].

The remaining six isolates that were sequenced all contained relatively minor changes but each change was sufficient to interrupt RPA primer binding or lead to excessively large fragments (Table 2). The numbers of RPA undetectable isolates in this study are consistent with those seen in previous studies on the PCR assays available on the market, and suggests that there will always be a few escapee isolates. However the amount of diversity seen here in just one hospital is likely to only be the tip of an iceberg and hence much larger studies of isolates from hospitals in different geographical areas are needed to understand the true levels of natural diversity amongst the populations of MRSA prevailing in our hospitals. The latest whole-genome sequencing technologies make this possible by multiplexing up to 192 samples in a single pool and we have shown that a *de novo* assembly approach is a reliable method to identify novel SCC*mec* element sequences.

To effectively reduce post-operative infection rates all surgical patients are screened for MRSA colonisation. In addition all medical admissions are also screened in most hospitals to reduce overall levels of colonisation and the risk of bacteraemia. Effective isolation and treatment of patients with MRSA colonisation requires adequate identification of resistant *S. aureus* and the speedier time to results that the molecular assays provide has to be balanced against their reduced sensitivity. However, clinical laboratories need to be cautious in adopting fully molecular assays at present – it is likely that 4–5% of MRSA will be missed. We suggest that further uptake occurs only in the knowledge that a phenotypic assay is used to confirm negative isolates. We await larger sequencing studies to provide more information on the ultra-conserved areas of the cassette that can be used for primer design.

## Supporting Information

Figure S1 Typical fluorescence (arbitrary units) curves for RPA *orfX*-SCC*mec* junction multiplex reactions. Fluorescence is generated by the cleavage of TwistAmp exo probes that have hybridised to amplicon produced by opposing primers. Primers are designed to amplify junction types i, ii, iii, iv, v and vii. a) Signal from the orfX probe (FAM) indicating the presence of the junction sequence. b) Signal from the internal control probe (TAMRA) showing that the reactions have worked. Two negative, no template, controls (NTC) were run. Reactions were run at 39°C for 20****minutes in Twista portable real-time fluorometers (www.twistdx.co.uk). The strips of 8**×**0.2 ml tubes were removed from Twista, agitated and replaced after 4 and 6****minutes – these are visible as spikes in fluorescence. Because RPA reactions are viscous and run at relatively low temperatures, agitation is necessary to disperse amplicons if there are not many starting template molecules.
(TIF)

Figure S2 Binary heatmap showing presence or absence of proteins within MRSA SCC*mec* elements. Presence or absence of proteins in SCCmec published sequences on GenBank using OrthoMCL to determine homology. Conventional gene names are shown for each orthologue group with uncharacterised (hypothetical) proteins listed with a relevant reference strain and locus tag in table S2.
(TIF)

Figure S3 Type II variant: homology between strains MRSA252 (NC_002952) and CMFT strain 2. **a)** Comparison between strain with novel SCC*mec* from CMFT and SCC*mec* type II reference strain shown on ACT (26) demonstrating very similar homology for the majority of the 3′ end of the cassette. **b)** Close-up view of 5′ end of SCC*mec* demonstrating main divergence with additional class 1 *ccr* complex inserted in CMFT2.
(TIF)

Figure S4 Type IVk: homology between strains CMFT201 and 45394F (GU122149). a) Comparison between strain with novel SCC*mec* from CMFT and SCC*mec* type IVk strain on GenBank focusing on the 3′ end, which demonstrates considerable homology. b) Close-up view of 5′ end of SCC*mec* demonstrating the only area of significantly reduced homology; with absence of *hsdR* and *hsdM* in CMFT201.
(TIF)

Figure S5 Type IVh variant SCC*mec* for CMFT3119 compared to strains ZH47 (AM292304) and M1 (HM030720). The cassette of CMFT3119 shows significant homology to ZH47 with the addition of an *arc* gene cluster similar to that found in M1.
(TIF)

Figure S6 Importance of assembly for examining SCC*mec* types in MRSA. **a)** VCF view on Artemis (27) for all 24 MRSA isolates sequenced (rows) with variants compared to MRSA252 shown as coloured vertical lines. Area shaded in pink is for SCC*mec* region. There appears to be little variation but **b)** is the same region in pink shown with a variation of varB (50) that displays areas of zero coverage as grey. The low variation seen in **a)** is due to a significant reduction in mapping depth over this region. **c)** BAM view of same reads of CMFT540 piled onto completed assembly of CMFT540 (only SCC*mec* is annotated).
(TIF)

Table S1 SCC*mec* element prototype strains used.
(DOCX)

Table S2 Locus tags for uncharacterised protein clusters in Figure S2.
(DOCX)
