# Supplementary figures and images for: Recombinations in Staphylococcal Cassette Chromosome mec Elements Compromise the Molecular Detection of Methicillin Resistance in Staphylococcus aureus
Source: PLoS One. 2014 Jun 27;9(6):e101419. doi: 10.1371/journal.pone.0101419 (PMC4074205; doi:10.1371/journal.pone.0101419)

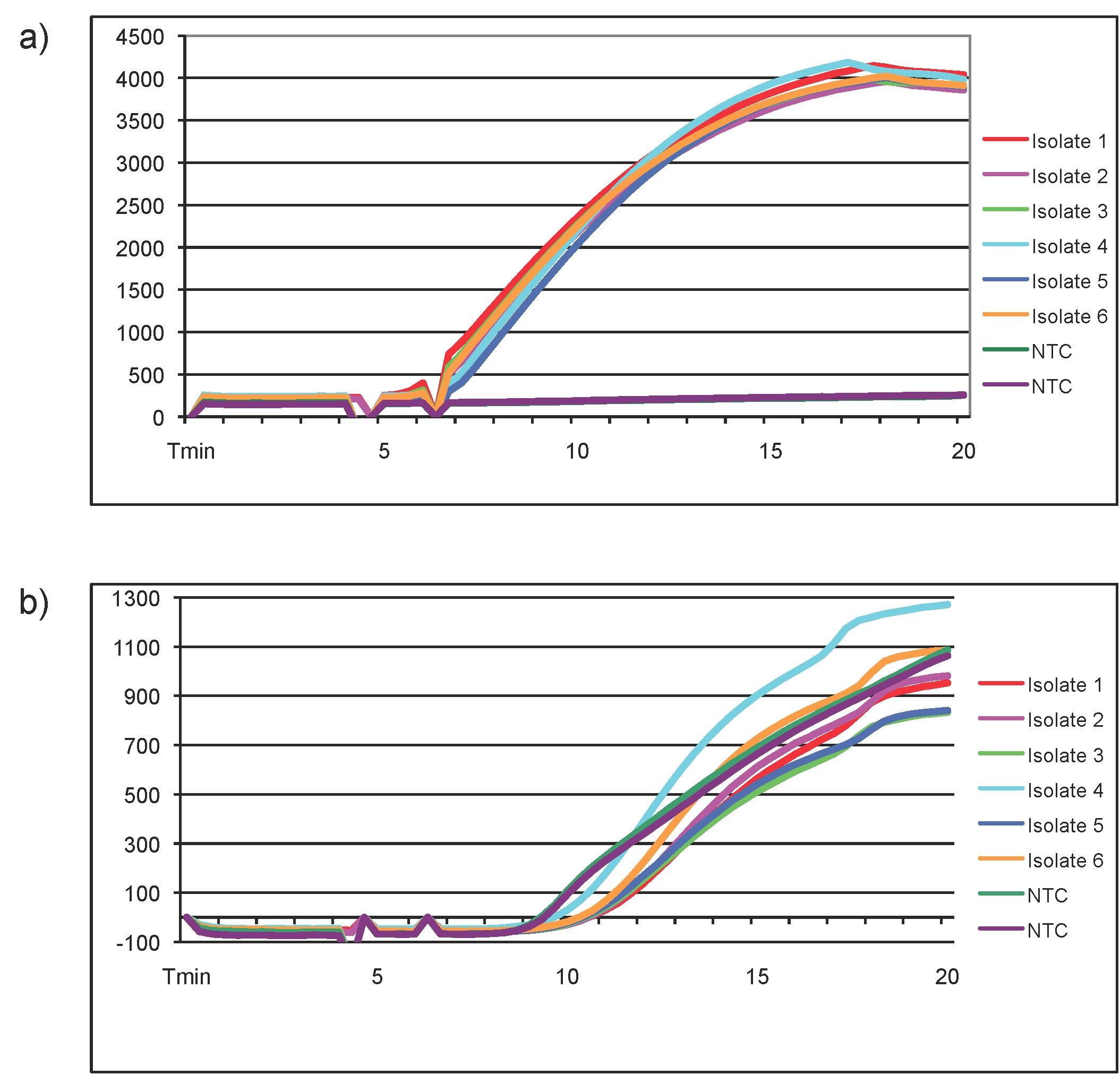

Supplement: Figure S1 — Typical fluorescence (arbitrary units) curves for RPA orfX-SCCmec junction multiplex reactions. Fluorescence is generated by the cleavage of TwistAmp exo probes that have hybridised to amplicon produced by opposing primers. Primers are designed to amplify junction types i, ii, iii, iv, v and vii. a) Signal from the orfX probe (FAM) indicating the presence of the junction sequence. b) Signal from the internal control probe (TAMRA) showing that the reactions have worked. Two negative, no template, controls (NTC) were run. Reactions were run at 39°C for 20minutes in Twista portable real-time fluorometers (www.twistdx.co.uk). The strips of 8×0.2 ml tubes were removed from Twista, agitated and replaced after 4 and 6minutes – these are visible as spikes in fluorescence. Because RPA reactions are viscous and run at relatively low temperatures, agitation is necessary to disperse amplicons if there are not many starting template molecules. (TIF) [file pone.0101419.s001.tif]

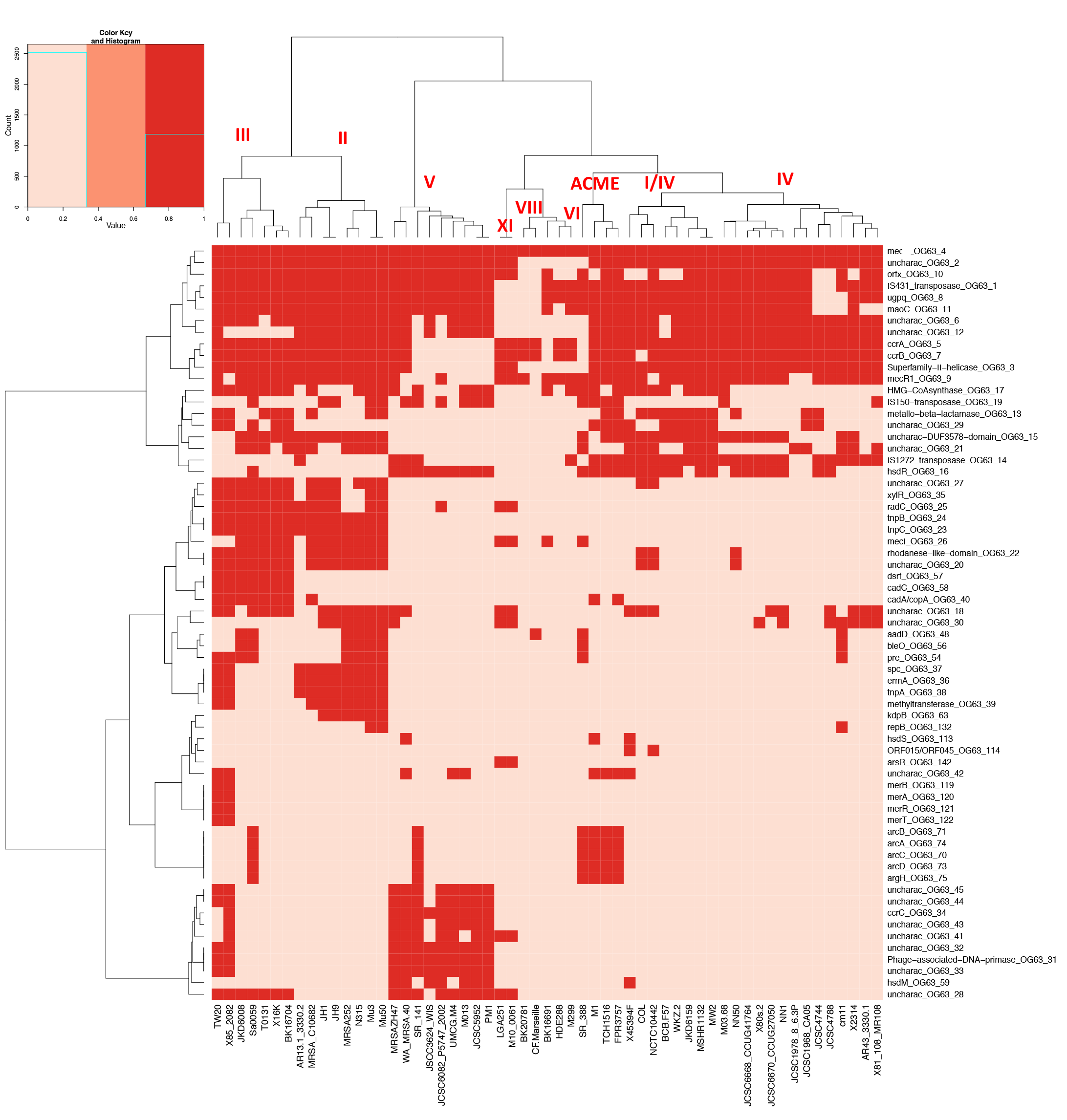

Supplement: Figure S2 — Binary heatmap showing presence or absence of proteins within MRSA SCCmec elements. Presence or absence of proteins in SCCmec published sequences on GenBank using OrthoMCL to determine homology. Conventional gene names are shown for each orthologue group with uncharacterised (hypothetical) proteins listed with a relevant reference strain and locus tag in table S2. (TIF) [file pone.0101419.s002.tif]

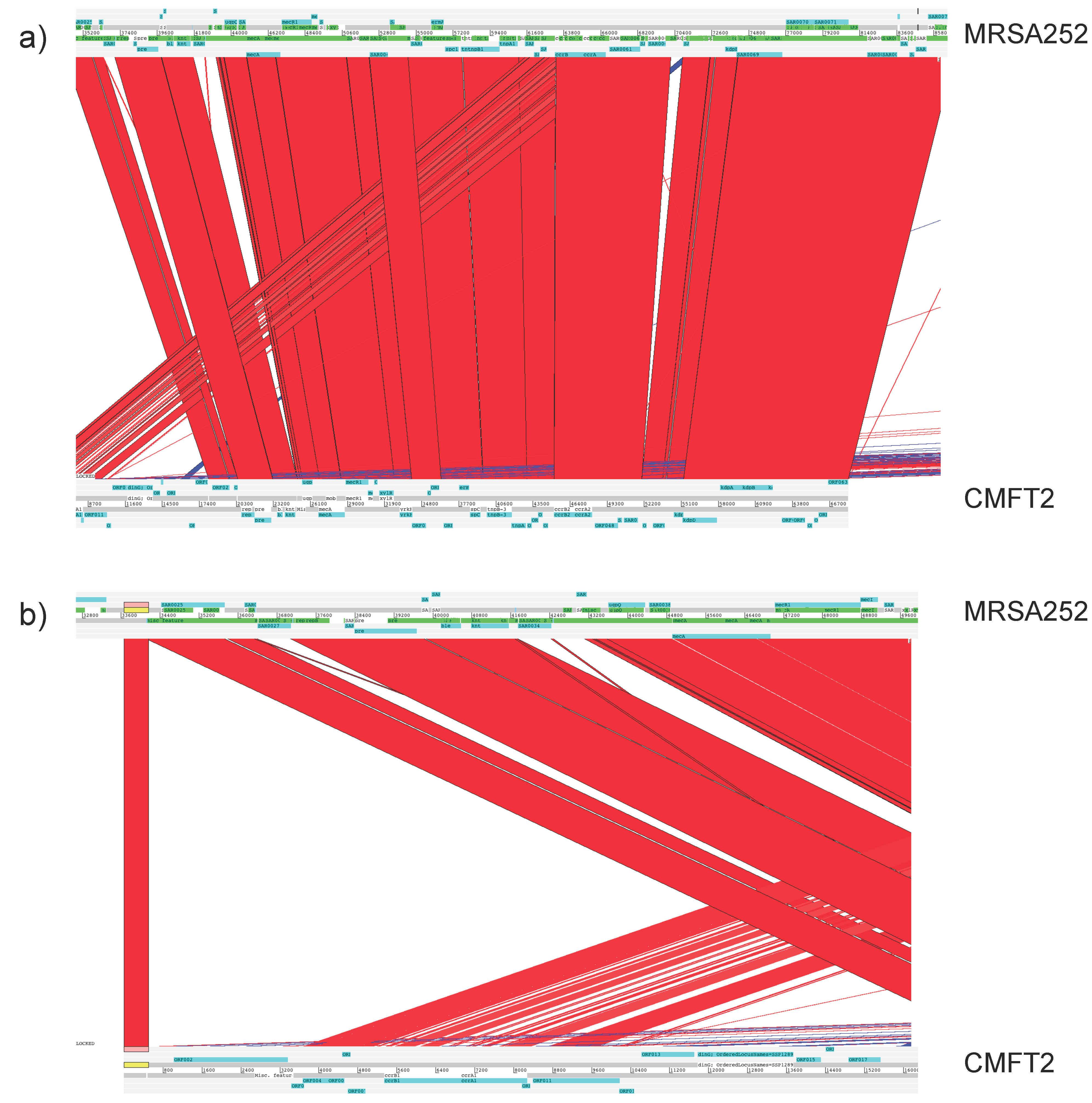

Supplement: Figure S3 — Type II variant: homology between strains MRSA252 (NC_002952) and CMFT strain 2. a) Comparison between strain with novel SCCmec from CMFT and SCCmec type II reference strain shown on ACT (26) demonstrating very similar homology for the majority of the 3′ end of the cassette. b) Close-up view of 5′ end of SCCmec demonstrating main divergence with additional class 1 ccr complex inserted in CMFT2. (TIF) [file pone.0101419.s003.tif]

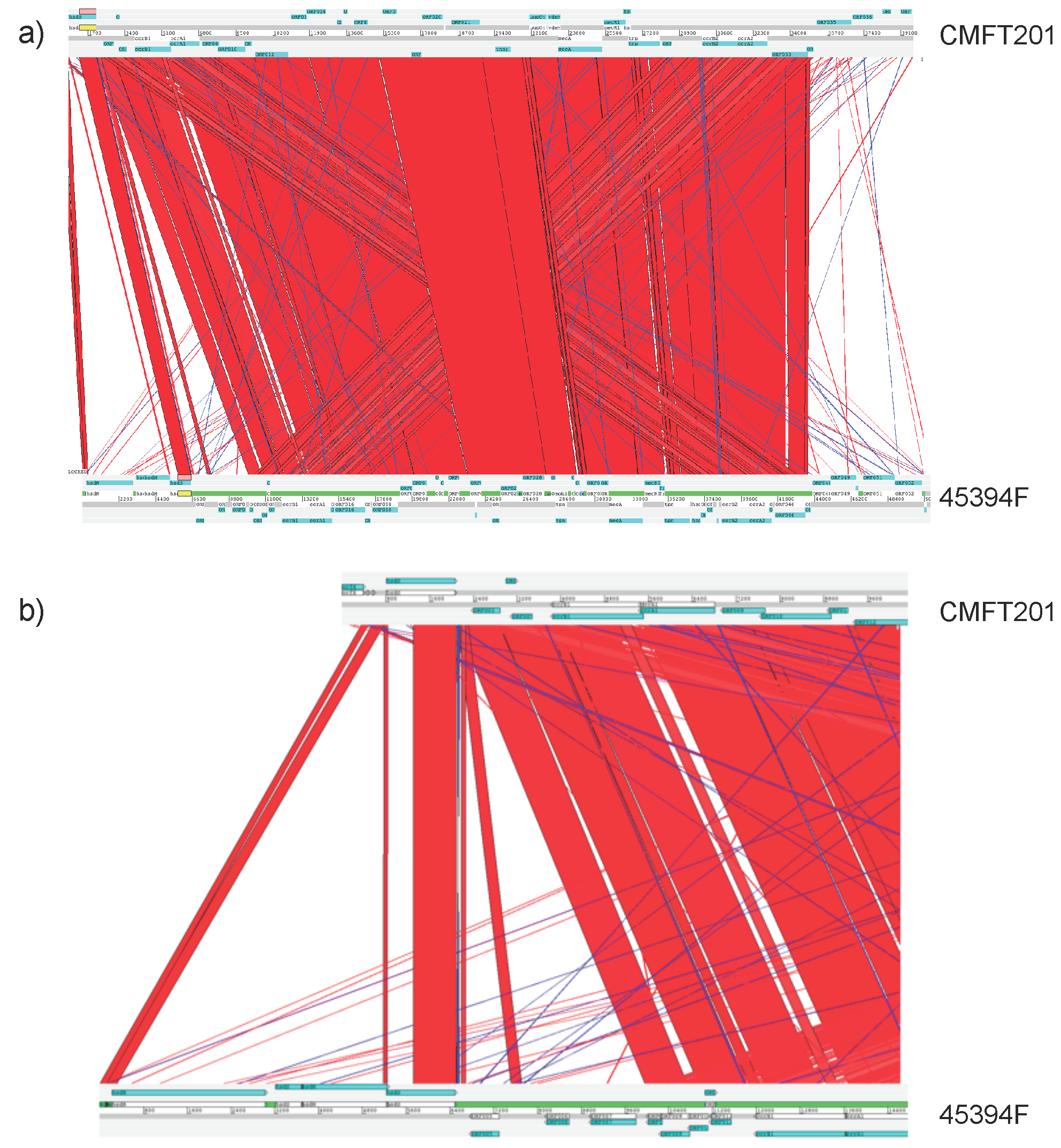

Supplement: Figure S4 — Type IVk: homology between strains CMFT201 and 45394F (GU122149). a) Comparison between strain with novel SCCmec from CMFT and SCCmec type IVk strain on GenBank focusing on the 3′ end, which demonstrates considerable homology. b) Close-up view of 5′ end of SCCmec demonstrating the only area of significantly reduced homology; with absence of hsdR and hsdM in CMFT201. (TIF) [file pone.0101419.s004.tif]

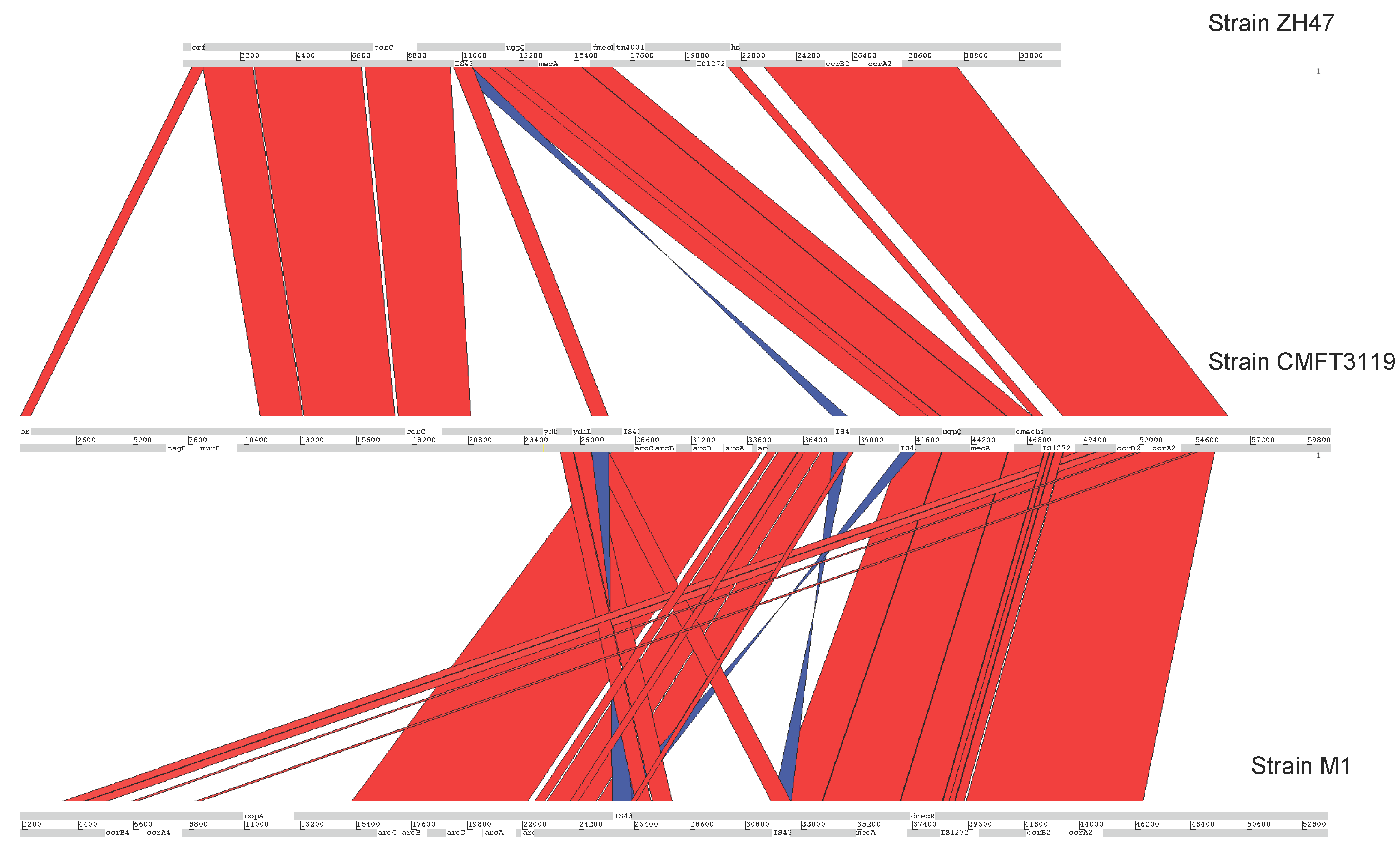

Supplement: Figure S5 — Type IVh variant SCCmec for CMFT3119 compared to strains ZH47 (AM292304) and M1 (HM030720). The cassette of CMFT3119 shows significant homology to ZH47 with the addition of an arc gene cluster similar to that found in M1. (TIF) [file pone.0101419.s005.tif]

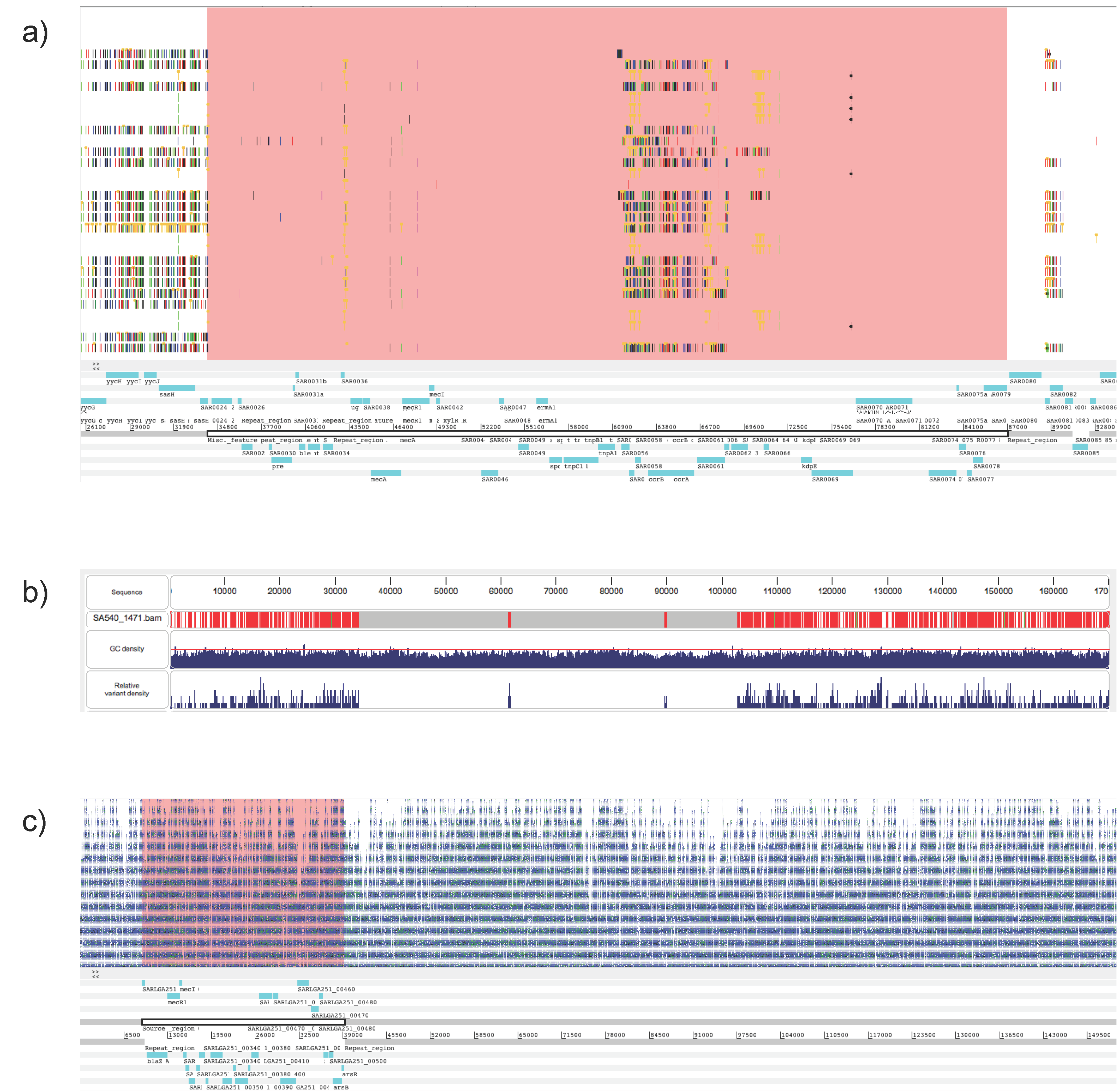

Supplement: Figure S6 — Importance of assembly for examining SCCmec types in MRSA. a) VCF view on Artemis (27) for all 24 MRSA isolates sequenced (rows) with variants compared to MRSA252 shown as coloured vertical lines. Area shaded in pink is for SCCmec region. There appears to be little variation but b) is the same region in pink shown with a variation of varB (50) that displays areas of zero coverage as grey. The low variation seen in a) is due to a significant reduction in mapping depth over this region. c) BAM view of same reads of CMFT540 piled onto completed assembly of CMFT540 (only SCCmec is annotated). (TIF) [file pone.0101419.s006.tif]
