# Supplementary material for: Recombinations in Staphylococcal Cassette Chromosome mec Elements Compromise the Molecular Detection of Methicillin Resistance in Staphylococcus aureus
Source: PLoS One. 2014 Jun 27;9(6):e101419. doi: 10.1371/journal.pone.0101419 (PMC4074205; doi:10.1371/journal.pone.0101419)
Supplement: Table S1 — SCCmec element prototype strains used. (DOCX) [file pone.0101419.s007.docx]

T**able S1. SCC*mec* element prototype strains used.**

| SCC*mec* type | Strain name(s)^a^ |
| --- | --- |
| I | NCTC10442, COL |
| II | N315, BK2464 |
| III | 85/2082, ANS46 |
| IVa | JCSC4744, MW2 |
| V | WIS |
| VI | HDE288 |
| VII | JCSC6082 |
| VIII | C10682 |
| IX | JCSC6943 |
| X | JCSC6945 |
| XI | LGA251 |

^a^ Strains were kindly provided by T. Ito, H. de Lencastre, B. Soderquist, K. Zhang, A. Larsen/R. Skov and M. Holmes.
