# Supplementary material for: Recombinations in Staphylococcal Cassette Chromosome mec Elements Compromise the Molecular Detection of Methicillin Resistance in Staphylococcus aureus
Source: PLoS One. 2014 Jun 27;9(6):e101419. doi: 10.1371/journal.pone.0101419 (PMC4074205; doi:10.1371/journal.pone.0101419)
Supplement: Table S2 — Locus tags for uncharacterised protein clusters in Figure S2. (DOCX) [file pone.0101419.s008.docx]

T**able S2. Locus tags for uncharacterised protein clusters in Figure S2.**

| **Cluster** | **Reference** | **Locus tag** |
| --- | --- | --- |
| OG63_2 | MRSA252 | SAR0056 |
| OG63_6 | MRSA252 | SAR0058 |
| OG63_12 | MRSA252 | SAR0057 |
| OG63_18 | MRSA252 | SAR0062 |
| OG63_20 | MRSA252 | SAR0047 |
| OG63_21 | MRSA252 | SAR0026 |
| OG63_27 | TW20 | SATW20_00810 |
| OG63_28 | TW20 | SATW20_00890 |
| OG63_29 | TW20 | SATW20_00960 |
| OG63_30 | MRSA252 | SAR0063 |
| OG63_31 | TW20 | SATW20_00320 |
| OG63_32 | TW20 | SATW20_00310 |
| OG63_33 | TW20 | SATW20_00300 |
| OG63_41 | LGA251 | SARLGA251_00350 |
| OG63_42 | TW20 | SATW20_00510 |
| OG63_43 | ZH47 | ZH04 |
| OG63_44 | TW20 | SATW20_00270 |
| OG63_45 | TW20 | SATW20_00260 |
